# The complete chloroplast genome of a medicinal resource plant (*Rumex crispus*)

**DOI:** 10.1080/23802359.2019.1660254

**Published:** 2019-08-30

**Authors:** Wei Tan, Han Gao, Tianying Yang, Weiling Jiang, Huanyu Zhang, Xiaolei Yu, Xiaoxuan Tian

**Affiliations:** Tianjin State Key Laboratory of Modern Chinese Medicine, Tianjin University of Traditional Chinese Medicine, Tianjin, China

**Keywords:** Chloroplast genome, *Rumex crispus*, medicinal plant

## Abstract

*Rumex crispus* has high medicinal value which belongs to the family Polygonaceae. We sequenced the complete chloroplast genome of *R. crispus*, which is 158,851 bp in length. A total of 111 unique genes have been predicted in the chloroplast genome of this species, consisting of 77 protein-coding sequences, 30 tRNA and 4 rRNA genes. A maximum likelihood (ML) phylogenetic tree based on 80 protein-coding genes of 18 Polygonaceae species showed the phylogenetic position of *R. crispus* within the family Polygonaceae. These results facilitate population and biological studies of *R. crispus* and benefit further investigations of this important species.

*Rumex crispus* is a perennial plant and belongs to the family Polygonaceae. *R. crispus* has a long history of domestic herbal use because of its high medicinal values. The roots, leaves and seeds of *R. crispus* have been reported to possess broad curative activities, including antioxidant, antimicrobial (Kim et al. 1999; Yildirim et al. 2001), hepatoprotective, anti-inflammatory and analgesic (Lee et al. 2007). Nevertheless, despite its importance, few studies have described the genome of *R. crispus*. Therefore, we establish and characterize the organization of the complete chloroplast genome of *R. crispus.* The results will also provide genetic information of this species as well as valuable knowledge for future research in the identification of Polygonaceae species.

Dried radix and rhizome powder of *R. crispus* (batch number 121291-201102) were collected from National Institutes for Food and Drug Control. Meanwhile, the specimens (voucher numbers TDH-2) were deposited in Tianjin State Key Laboratory of Modern Chinese Medicine, Tianjin, China. Total genomic DNA was extracted using Extract Genomic DNA Kit with a standard protocol. The DNA was sequenced using the Illumina Hiseq X Ten platform with 800 bp insert size. The clean data was *de novo* assembled using NOVOPlasty v3.1 (Dierckxsens et al. 2017) and GetOrganelle pipeline (Jin et al. 2018). Then, the resulting contigs were identified and rearranged by mapping to reference genome *Rumex acetosa* (Gui et al. 2018) (Accession number: MH359405). Annotation of the chloroplast genome was performed using CpGAVAS2 (Shi et al. 2019) and GeSeq (Tillich et al. 2017). Subsequently, the annotation of two methods above was compared and inspected manually. The tRNAscan-SE search server (Lowe and Eddy 1997) was employed to verify the annotations of tRNA genes. Finally, the physical genome map was illustrated with OGDraw (Greiner et al. 2019), and the complete annotated plastome of *R. crispus* was deposited into GenBank (accession numbers: MN055629).

In this study, the complete chloroplast genome presented 158,851 bp in size, with a total of 111 unique genes including 77 protein-coding sequences, 30 tRNA and 4 rRNA genes. Among these genes, 15 genes (*rpl16, rpl2, rps16, rpoC1, trnA-UGC, trnG-UCC, trnI-GAU, trnK-UUU, trnL-UAA, trnV-UAC, ndhA, ndhB, petB, petD,* and *atpF*) contained one intron and two genes (*clpP* and *ycf3*) harbored two introns.

To infer the phylogenetic position of *R. crispus* within the family Polygonaceae, 17 Polygonaceae species plastomes were downloaded from the Genbank database. All 80 protein-coding genes were extracted and aligned using MAFFT v7 (Katoh and Standley 2013) with default parameters and concatenated to a final data matrix. RAxML v8 (Stamatakis 2014) was employed to reconstruct the maximum-likelihood (ML) phylogeny based on the GTRGAMMA model using 1000 replicates of bootstrap analysis. In the maximum-likelihood (ML) tree, most of the nodes had a 100% bootstrap value. *Rumex* exhibited the closest relationship with other two genera, *Rheum* and *Oxyria.* Meanwhile, the phylogenetic positions of *Rumex* were in agreement with recent studies (Gao et al. 2019) (Figure 1). Overall, the results of this study will help to provide valuable resources for molecular biology and evolutionary studies of *R. crispus*.

**Figure 1.**
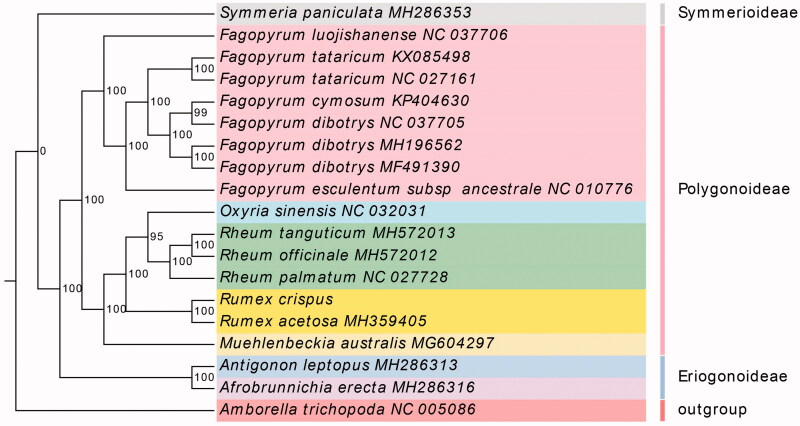
Maximum-likelihood phylogenetic tree constructed with 80 protein-coding genes of 19 species. Numbers at nodes are values for bootstrap support.
